# Stromal Cell Signature Associated with Response to Neoadjuvant Chemotherapy in Locally Advanced Breast Cancer

**DOI:** 10.3390/cells8121566

**Published:** 2019-12-04

**Authors:** Maria Lucia Hirata Katayama, René Aloísio da Costa Vieira, Victor Piana Andrade, Rosimeire Aparecida Roela, Luiz Guilherme Cernaglia Aureliano Lima, Ligia Maria Kerr, Adriano Polpo de Campos, Carlos Alberto de Bragança Pereira, Pedro Adolpho de Menezes Pacheco Serio, Giselly Encinas, Simone Maistro, Matheus de Almeida Leite Petroni, Maria Mitzi Brentani, Maria Aparecida Azevedo Koike Folgueira

**Affiliations:** 1 Departamento de Radiologia e Oncologia, Centro de Investigação Translacional em Oncologia, Instituto do Cancer do Estado de Sao Paulo, Hospital das Clinicas HCFMUSP, Faculdade de Medicina, Universidade de Sao Paulo, Sao Paulo 01246-000, SP, Brazil; maria.katayama@fm.usp.br (M.L.H.K.); r.roela@fm.usp.br (R.A.R.); pedro.serio@fm.usp.br (P.A.d.M.P.S.); gisellyencinas@yahoo.com.br (G.E.); simone.maistro@hc.fm.usp.br (S.M.); malpetroni@gmail.com (M.d.A.L.P.); m.brentani@fm.usp.br (M.M.B.); 2 Departamento de Mastologia, Hospital de Câncer de Barretos, Barretos 14.784-400, SP, Brazil; reneacv@terra.com.br; 3 A.C. Camargo Cancer Center, Sao Paulo 01525-001, SP, Brazil; victorpiana@me.com (V.P.A.); luiz_calima@yahoo.com.br (L.G.C.A.L.); 4 Departamento de Patologia, Hospital de Câncer de Barretos, Barretos 14.784-400, SP, Brazil; wduzzi@yahoo.com.br; 5 Departamento de Estatística, Centro de Ciências Exatas e de Tecnologia, Universidade Federal de São Carlos, Sao Carlos 13565-905, SP, Brazil; polpo@ufscar.br; 6 Department of Mathematics and Statistics, The University of Western Australia, M019, 35 Stirling Highway, 6009 Crawley, WA, Australia; 7 Departamento de Estatística, Instituto de Matemática e Estatística, Universidade de São Paulo, São Paulo 05508-090, SP, Brazil; cadebp@gmail.com

**Keywords:** breast cancer, stromal cells, gene expression, chemotherapy neoadjuvant

## Abstract

Breast cancer stromal compartment, may influence responsiveness to chemotherapy. Our aim was to detect a stromal cell signature (using a direct approach of microdissected stromal cells) associated with response to neoadjuvant chemotherapy (neoCT) in locally advanced breast cancer (LABC). The tumor samples were collected from 44 patients with LABC (29 estrogen receptor (ER) positive and 15 ER negative) before the start of any treatment. Neoadjuvant chemotherapy consisted of doxorubicin and cyclophosphamide, followed by paclitaxel. Response was defined as downstaging to maximum ypT1a-b/ypN0. The stromal cells, mainly composed of fibroblast and immune cells, were microdissected from fresh frozen tumor samples and gene expression profile was determined using Agilent SurePrint G3 Human Gene Expression microarrays. Expression levels were compared using MeV (MultiExperiment Viewer) software, applying SAM (significance analysis of microarrays). To classify samples according to tumor response, the order of median based on confidence statements (MedOr) was used, and to identify gene sets correlated with the phenotype downstaging, gene set enrichment analysis (GSEA). Nine patients presented disease downstaging. Eleven sequences (FDR 17) were differentially expressed, all of which (except *H2AFJ*) more expressed in responsive tumors, including *PTCHD1* and genes involved in abnormal cytotoxic T cell physiology, *TOX*, *LY75*, and *SH2D1A*. The following four pairs of markers could correctly classify all tumor samples according to response: *PTCHD1/PDXDC2P*, *LOC100506731/NEURL4*, *SH2D1A/ENST00000478672*, and *TOX/H2AFJ*. Gene sets correlated with tumor downstaging (FDR < 0.01) were mainly involved in immune response or lymphocyte activation, including CD47, LCK, NCK1, CD24, CD3E, ZAP70, FOXP3, and CD74, among others. In locally advanced breast cancer, stromal cells may present specific features of immune response that may be associated with chemotherapy response.

## 1. Introduction

Neoadjuvant chemotherapy (neoCT) has a central role in the management of locally advanced breast cancer and a number of different response predictive signatures have been identified, however, to the present moment, none of them is used in clinical practice [1,2]. In common, these expression profiles are derived from the whole tumor tissue that comprehends both malignant cells, as well as variable proportions of stromal cells.

Breast cancer behavior is a reflection of an interactive signaling between the malignant epithelial compartment and the surrounding microenvironment, composed of stromal cells, including carcinoma-associated fibroblasts, mesenchymal stem cells, tumor-associated macrophages, endothelial cells, pericytes, adipocytes, and lymphocytes, as well as extracellular matrix components [3]. The interactions among these compartments may be mediated by secreted factors, cell–matrix interactions, as well as cell-cell direct contact.

There is evidence that stromal cells of normal tissues behave quite distinctly from stromal cells of tumoral tissues. Normal stroma may inhibit cell proliferation, in contrast with tumor stroma that may support tumor development and progression, through the induction of cancer cells proliferation, migration, and invasion, as well as the activation of angiogenesis [3,4,5]. There is also evidence that both the extracellular matrix, as well as the stromal cells, may play a role in drug resistance and disease prognosis [6,7,8]. Accordingly, there have been reports indicating that the disorganized stroma is associated with poor response to chemotherapy but, in contrast, it is inversely related with lymph node metastases [9]. In addition, it has been suggested that high stromal gene expression, characterizing a reactive stroma, may be associated with resistance to neoadjuvant chemotherapy in estrogen receptor negative tumors [7]. This signature, however, was not specifically derived from the stromal tumor compartment itself. Furthermore, in triple negative breast cancer, increased levels of stromal tumor infiltrating lymphocytes have been shown to predict pathologic complete response [10,11].

Hence, our aim was to evaluate whether a stromal cell transcriptional signature might be associated with response to neoadjuvant anthracycline and taxane, in locally advanced breast cancer, using a direct approach of stromal cell selection.

## 2. Patients and Methods

This protocol was approved by the Institutional Ethics Committee (Comitê de Ética do Hospital de Câncer de Barretos, protocol number 135/2008; Comitê de Ética para Análise de Projetos de Pesquisa do Hospital das Clínicas da Faculdade de Medicina da Universidade de São Paulo, protocol number 1256/09). This study was registered at ClinicalTrials.gov (Identifier NCT00820690). A written informed consent was signed by all participants.

Inclusion criteria were women with locally advanced breast cancer and clinical conditions to receive treatment with doxorubicin, cyclophosphamide, and paclitaxel. Exclusion criteria were patients diagnosed with inflammatory breast cancer or previous treatment for breast cancer. All tumor samples were collected before the start of neoadjuvant chemotherapy.

Neoadjuvant chemotherapy followed the hospital treatment protocol, consisting of 4 cycles of doxorubicin 60 mg/m^2^ and cyclophosphamide 600 mg/m^2^, every 21 days, followed by 4 cycles of paclitaxel 174 mg/m^2^ every 21 days (or 80 mg/m^2^ weekly for 12 weeks). Response was defined as pathological complete response (PCR) or downstaging to maximum ypT1a-b/ypN0, after chemotherapy.

### Stromal Cell Selection and Microarray Analysis

Fresh frozen tumor fragments collected before any treatment were cut in 10 µm slices. Intratumoral stroma, mainly comprising fibroblasts, sometimes accompanied by immune cells was microdissected using CapSure HS LCM (Thermo Fisher Scientific, Waltham, MA, USA) in a Pix Cell II Arcturus Laser Capture Microdissection (Thermo Fisher Scientific) (LCM) (Figure S1). Tumor nests and blood vessels were avoided. After addition of a lysis solution, total RNA was recovered from RNA purification column (Arcturus PicoPure RNA isolation, Thermo Fisher Scientific).

Both total RNA extracted from samples and Universal Reference RNA (Stratagene California, La Jolla, CA, USA) were linearly amplified, using two-round RiboAmp HS^Plus^2-round (Thermo Technologies) and Low Input Quick Amp kit (Agilent Technologies, Santa Clara, CA, USA) protocols and labelled with CY5 or CY3, respectively. Samples were analyzed on a NanoDrop spectrometer (Thermo Fisher Scientific) and yield values varied between 0.79 and 5.93 µg of cRNA and specific activity between 6.36 and 10.75 pMol Cy3 or Cy5 per µg cRNA. Competitive hybridization was performed in the SurePrint G38×60K slides (Agilent Technologies) and fluorescence intensities from scanned image files in an Agilent Bundle Model B Microarray Scanner System (Agilent Technologies) were preprocessed with Agilent Feature Extraction software (v10.7.1) and normalized using GeneSpring GX12.1 software (Agilent Technologies). A detailed description of methods is provided in Supplementary methods.

Comparisons of expression levels were performed using MeV (MultiExperiment Viewer, version 4.5.1.) software applying SAM (significance analysis of microarrays). Analysis was done using all sequences (without filtering). Unsupervised hierarchical clustering based on Euclidean distance and average linkage was used to verify association patterns. The reliability of the clustering was assessed by bootstrap technique using MeV. Raw data from microarray was deposited at the Gene Expression Omnibus (GEO).

Classification of stromal samples according to response was also analyzed using the order of medians, based on confidence statements [12,13]. This method compares the median expression of each microarray marker for detection of a difference between the two populations of samples (downstaging vs. non-downstaging), and a confidence statement of the difference. With the list of confidence statements for all markers, pairs of over- and underexpressed markers are chosen sequentially, based on the highest confidence values. The first pair is chosen as the markers over- (O) and underexpressed (U) in downstaging (as compared with non-downstaging) samples with the highest confidence; the relation index O/U is calculated for each sample. Afterwards, the confidence statement that median index value for O/U, is greater in downstaging (M1) as compared with non-downstaging samples (M2), is computed. If the confidence statement is not high enough, the second pair of over- and underexpressed genes (with the second highest confidence) is added. Then, the product of the two overexpressed markers is divided by the product of the two underexpressed, for each sample, and a new confidence statement is calculated for M1 > M2, and if not high enough, a third pair of markers is added, and so on, until a final index value with a satisfactory confidence is attained for the separation of the two populations of samples (Supplementary methods) [12,13].

Gene list enrichment analysis and candidate gene prioritization, based on functional annotations and protein interactions network, was performed using Toppgene suite, with FDR correction *p* value cut off ≤ 0.05 (https://toppgene.cchmc.org/enrichment.jsp) [14].

Gene set enrichment analysis (GSEA) was used to identify whether predefined gene sets might associate with gene expression differences between phenotypes (available at http://software.broadinstitute.org/gsea/index.jsp). In this pairwise comparison, all genes are ranked based on signal-to-noise ratio. Then, the alternative hypothesis, that rank ordering of distinct pathway members is associated with a specific phenotype, is tested [15]. This methodology makes it possible to detect situations where all genes, in a predefined set, change in a small, but coordinated way. FDR < 0.25 was considered significant. Some results assumed FDR < 0.1 or < 0.01, because even using more stringent cut offs, these lists comprehended at least 100 gene sets. The GSEA collection was identified by searching google tool for gene set name.

Data derived from gene expression was also investigated for enriched networks using Ingenuity Pathway Analysis, IPA (Qiagen).

The ROC plotter, an online transcriptome-level validation tool for predictive biomarkers, was used to investigate the potential association of 10 highlighted genes with pathological complete response to any chemotherapy [16].

## 3. Results

### 3.1. Patients

Forty-four patients diagnosed with locally advanced breast cancer, between July 2008 and January 2012, at the Hospital de Câncer de Barretos, Barretos, SP, Brazil, were included. Patients’ median age was 43 years (21–64 y). All patients presented stage III disease and mean tumor dimension prechemotherapy was 7.0 cm (± 2.0) and postchemotherapy was 4.2 cm (± 3.4). All patients, except for three, were diagnosed with invasive ductal carcinoma and among tumors, 29 were classified as estrogen receptor (ER) positive and 15 were classified as ER negative (Table 1). All patients received the recommended neoadjuvant chemotherapy, except for five, who interrupted treatment due to intolerance or lack of tumor reduction. Median time between last cycle of chemotherapy and breast surgery was 35 days. After chemotherapy, nine patients presented disease downstaging to maximum ypT1a-b/ypN0, including four, who presented pathological complete response. After a median follow up of 60 months (9.0–87.0 months), 23 patients presented recurrence of the disease, among whom, 20 patients died due to cancer. Another patient died from a cause other than cancer. All 44 patients had their pre neoCT sample collected

### 3.2. Stromal Cells Transcriptional Profile

To characterize the microdissected samples, we selected the top 50 highly expressed genes among all samples and performed gene list enrichment analysis (Toppgene Suite analysis) [14]. These genes were mainly involved in extracellular matrix structural constituent (COL3A1, COL10A1, CILP, ASPN, and FGL2); regulation of immune system process (COL3A1, SLAMF8, CXCL14, CCL19, FGR, CD14, FCGR3A, HLA-DOA, MNDA, HLA-DQA2, FPR3, PIP, and FGL2); and collagen-containing extracellular matrix (COL3A1, SPARCL1, COL10A1, SMOC2, CILP, ASPN, WNT2, F13A1, EGFL6, and FGL2) (Table S1).

**Figure 1:**
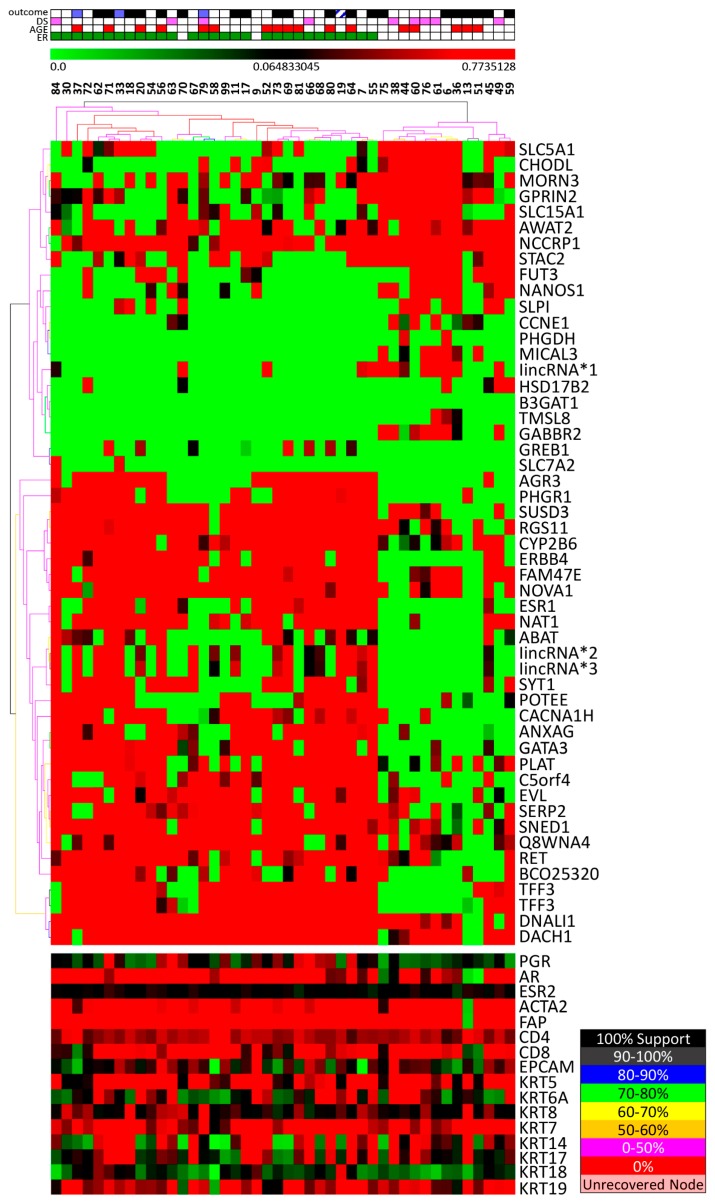
Unsupervised hierarchical clustering of stromal cells microdissected from tumors categorized according to estrogen receptor status (determined by immunohistochemistry of FFPE tumor fragment and shown in the upper panel). Estrogen receptor (ER) positive marked in green. The ER and progesterone receptor (PR) expression in malignant cells were evaluated using anti-estrogen receptor alpha rabbit monoclonal antibody SP1 (Thermo Fisher Scientific, Walthan, MA, USA) and CONFIRM antiprogesterone receptor rabbit monoclonal antibody (Roche AB, Christian Sundberg, Stockholm, Sweden), respectively, and were considered positive if ≥1% malignant cells were stained. Stromal cells were microdissected from samples. The gene expression profile was determined using Agilent platform and 51 sequences were found differentially expressed. Each column represents one tumor sample and each line represents the expression of one sequence (green less expressed and red, more expressed). Gene symbol appears on the right. Lines on the top of the dendogram show: black, blue, green indicate high confidence; yellow and pink indicate low confidence (color scale in accordance to support is represented in the box, on the right). Characteristics of patients and tumor samples appear on the upper box: outcome (blue, alive with disease recurrence and black, deceased); age (red, ≤40y); ER immunohistochemistry (green, positive); DS (downstaging) (pink, yes). Green (more expressed) and red (less expressed) scale bar on the top.

We then selected some classical fibroblast, lymphocyte, and epithelial markers to evaluate their expression, which is shown on Figure 1. On the one hand, overall, among all samples, there was a relative high expression of *ACTA2* (smooth muscle α actin) and *FAP* (fibroblast activation protein alpha), that are expressed by myofibroblasts and by reactive stromal fibroblasts of epithelial cancers (https://www.ncbi.nlm.nih.gov/gene/2191) [17], respectively, as well as CD4 and CD8, that are expressed by lymphocytes. On the other hand, expression of basal and luminal keratins was variable among microdissected samples and was not different between luminal (ER positive) and triple negative tumors, except for KRT18, which was more expressed in luminal tumors (Table S2). These results indicate that these microdissected samples were enriched in fibroblasts and immune cells.

In addition, we evaluated whether estrogen receptor status, (determined through immunohistochemistry in the FFPE tumor fragment) would classify the microdissected stromal cells. Using SAM test (FDR zero), 51 sequences were differentially expressed, 32 more expressed in stromal cells from ER positive (including *ESR1*, *GATA3*, *NAT1*, *TFF3*), and 19 more expressed in ER negative samples (Table S3). Unsupervised hierarchical clustering and bootstrapping, using these sequences, could separate samples in two branches, one of them including all 29 ER positive (by immunohistochemistry) and the other including 13/15 ER negative samples (Figure 1), resulting in 95.4% accuracy. Gene list enrichment analysis (Toppgene Suite analysis), showed that differentially expressed genes were classified as “genes upregulated in breast cancer samples positive for ESR1 as compared with the ESR1 negative tumors” such as: *ABAT*, *ANXA9*, *DACH1*, *DNALI1*, *ERBB4*, *ESR1*, *EVL*, *GATA3*, *GREB1*, *NAT1*, *RET*, *SYT1*, and *TFF3*.

The bottom expression box shows the expression of selected hormone receptors, as well as fibroblast, lymphocyte, and epithelial cell markers.

Additionally, we further explored the relative gene expression of other important hormonal receptors in breast cancer, such as estrogen receptor beta (ESR2), progesterone receptor (PGR) and androgen receptor (AR), concomitantly with estrogen receptor alpha (ESR1) [18,19]. In stromal cells, only the expression of ESR1 and PGR were correlated (r = 0.39, *p* = 0.0092, Pearson correlation), in contrast with AR vs. ESR1, AR vs. PGR, AR vs. ESR2, ESR1 vs. ESR2, and PGR vs. ESR2, which were not correlated (Table S4). Furthermore, AR was more expressed in ER positive samples (IMH of FFPE samples) as compared with triple negative samples (*p* = 0.0052, Mann Whitney test).

Then, we evaluated whether information on the age of the patients, categorized as ≤40 years (young adults) and >40 years, would classify the microdissected stromal cells. A differential gene expression profile, according to the patients´ ages, included 30 sequences (SAM, FDR 10) (Table S5), all of which were more expressed in patients older than 40 years. Hierarchical clustering, using this gene expression profile, correctly classified 93.1% of the samples (Figure S2), however, no gene ontology categories, enriched in these differentially expressed genes, were identified (http://toppgene.cchmc.org/enrichment.jsp).

**Figure 2:**
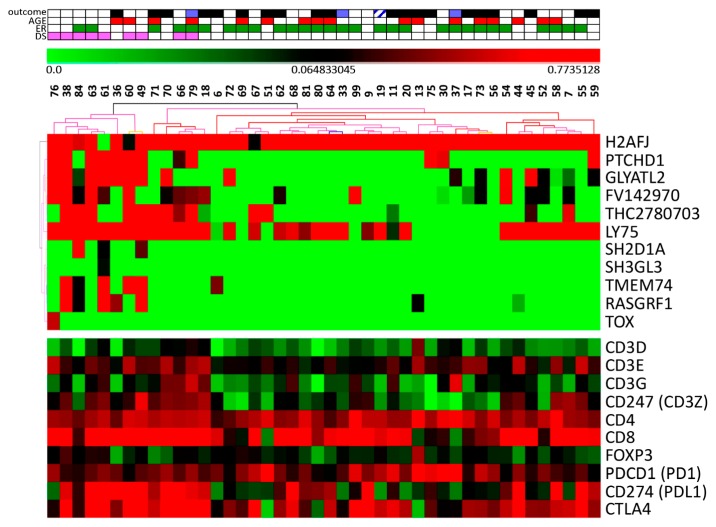
Unsupervised hierarchical clustering of stromal cells from tumors categorized as DS (downstaging) and NDS (non-downstaging). Tumor samples were collected before neoCT and stromal cells were microdissected. The gene expression profile was determined using SurePrint G3 Microarray, Agilent, applying SAM test (FDR 17) and 11 sequences were found differentially expressed between DS vs. NDS. Unsupervised hierarchical clustering and bootstrapping, using these sequences, identified two branches with high confidence, one including 9/44 downstaging (DS in pink, upper box) samples and the other including all non-downstaging (non-responsive) samples and one responsive sample, as well. Green (more expressed) and red (less expressed) scale bar on the top.The bottom expression box shows the expression of selected immune cell markers.

Our next aim was to identify a differential gene expression profile that characterized tumor downstaging (to at least ypT1a/b, ypN0). Using SAM test (FDR 17), 11 sequences (including nine genes) were differentially expressed, all of which (except for *H2AFJ*) more expressed in responsive tumors, from patients who presented tumor downstaging (Table S6). These sequences could correctly classify 93.1% of the samples, with high confidence, using unsupervised hierarchical clustering and bootstrapping (Figure 2). Gene list enrichment analysis revealed that three genes involved in “abnormal cytotoxic T cell physiology (mouse phenotype)”: *TOX*, *LY75*, and *SH2D1A* (http://toppgene.cchmc.org/enrichment.jsp) were more expressed in responsive samples. In addition, ingenuity pathway analysis, IPA, revealed a network enriched in the gene list “cancer” (Figure S3).

**Figure 3:**
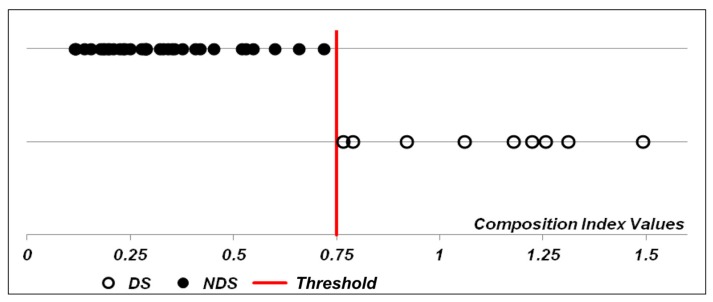
Classification of stromal cells samples according to tumor response (downstaging, DS, vs. non-downstaging, NDS) according to the order of median based on confidence statements. Sample classification was based on the composition index value, calculated as the product of the relation of pairs of genes overexpressed and underexpressed in downstaging samples: PTCHD1 and PDXDC2P, LOC100506731 and NEURL4, SH2D1A and ENST00000478672, and TOX and H2AFJ. The red line represents the threshold to classify samples in one of the two groups.

To further evaluate genes that might classify samples according to tumor response we used another statistical method, the order of median based on confidence statements. This method allows the identification of pairs of markers that may classify downstaging vs. non-downstaging samples. At first, median expression for each microarray marker was compared in downstaging vs. non-downstaging samples and the confidence of difference between medians was computed. With a list of 446 microrarray markers with confidence statements >0.95, over- and underexpressed pairs of markers in downstaging samples, based on the highest confidence statements, were chosen sequentially, and the relation between them was calculated for each sample. For our dataset, we could stop at the following four pairs of markers that were over- and underexpressed, respectively, in downstaging samples: *PTCHD1/PDXDC2P, LOC100506731/NEURL4*, *SH2D1A/ENST00000478672*, and *TOX/H2AFJ*. The final index, representing the product of the relation of these four pairs of markers, could separate each tumor sample, according to response to neoadjuvant chemotherapy, and values appear in Figure 3. This final index allowed us to state that the confidence that the median value in downstaging sample was greater than in non-downstaging samples, Mi_1_ > Mi_2_, was 99.8.

**Figure 4:**
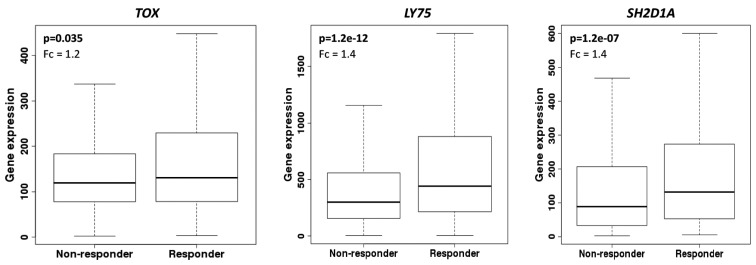
Expression of biomarkers according to pathological complete response. ROC plotter analysis (Fekete et al., 2019 [16]) considering 1632 samples from breast cancer patients (1100 non-responders, 532 responders) to any neoadjuvant chemotherapy. P, Mann–Whitney test and Fc, fold change. Probes used for TOX: 204529_s_at, Ly75: 205668_at, and SH2D1A: 210116_at.

Moreover, we evaluated the predictive potential of our main candidates, using ROC plotter, an on line tool that allows assessment to publicly available transcriptomic results of a large set of breast cancer patients submitted to neoadjuvant chemotherapy, that includes: taxane, anthracycline, ixabepilone, CMF, FAC, and FEC. Interestingly, all three genes involved in “abnormal cytotoxic T cell physiology (mouse phenotype)”, *TOX*, *LY75*, and *SH2D1A*, were more expressed in responsive samples, confirming our results (Figure 4). However, *PTCHD1, H2AFJ*, and *NEURL4* were not differentially expressed in this set of samples.

Our next step was to use GSEA to identify gene sets that were correlated with the phenotype downstaging or with the phenotype non-downstaging. Considering gene expression derived from all 44 tumor samples, GSEA revealed that 117 gene sets correlated with tumor downstaging (FDR < 0.01), including seven KEGG gene sets, comprehending two immune system-related gene sets (antigen processing and presentation, T cell receptor signaling pathway), as well as 15 GO biological process gene sets among which, at least 10, involved with immune response, including five implicated in induction or activation of this process: positive regulation of lymphocyte activation, positive regulation of immune system process, positive regulation of T cell activation, T cell activation, and lymphocyte activation (Table 2; Table S7). In addition, GSEA revealed 23 gene sets correlated with tumor non-downstaging (FDR < 0.25), none of them cataloged in the KEGG pathway database and only one cataloged in the GO gene set (cellular component) synaptic vesicle (Table S8).

As many gene sets associated with tumor downstaging were related with immune response, we evaluated expression of some classical T cell markers individually (using the Mann–Whitney test) in this group of microdissected samples. CD8, CD3E, and CD247 (CD3Z) were all more expressed in DS samples, whereas CD4, CD3D, and CD3G were not differentially expressed between DS and NDS. In addition, markers of an immunosuppressive microenvironment, such as FOXP3, CD274 (PDL1), and CTLA4 were also more expressed in responsive tumors (Figure 2 and Table S9). However, it is important to point out that these genes were not differentially expressed considering FDR in the SAM analysis of the microarray, which means that this analysis is not robust.

In addition, we evaluated whether the stromal mRNA expression of hormone receptors might be predictive of tumor response. Nonetheless, ESR1, ESR2, PGR, and AR were not differentially expressed in DS vs. NDS samples (Table S9).

We also used GSEA to identify gene sets that correlated with tumor response specifically in ER positive tumors, as well as in ER negative tumors, separately.

In ER positive tumors, the GSEA revealed 214 gene sets (FDR < 0.25) that correlated with tumor downstaging (Table S10). Using a more stringent FDR value (<0.1), tumor downstaging was correlated with one KEGG gene set, i.e., antigen processing antigen presentation (comprehending *CD74*, *CD8A*, *CD8B*, and nine molecules of HLA complex, among others) and four GO gene sets (biological process), including one related with immune response, i.e., regulation of T cell activation (comprehending genes such as *ZAP70*, *LCK*, *CD24*, *CD47*, and *CD3E*, among others).

In ER negative samples, the GSEA identified 998 gene sets, using FDR < 0.25, that were correlated with tumor downstaging. Using a more stringent FDR cut off (<0.05), five KEGG gene sets were correlated with tumor downstaging, including two related ith immune response, i.e., antigen processing and presentation and T cell receptor signaling pathway, as well as at least 12 GO gene sets (biological process) related with the immune process, including two with FDR < 0.01, which were adaptive immune response and positive regulation of T cell activation (Table S11).

In ER positive samples that did not present tumor downstaging, 49 gene sets were found in the GSEA analysis (FDR < 0.25) including one KEGG gene set, i.e., ECM receptor interaction (including five types of integrins, six collagens, four laminins, one fibronectin, and one trombospondin); eight GO gene sets; (biological process) cell adhesion; cell recognition; embryonic development; (cellular component) cortical cytoskeleton; cell projection part; cell surface; cytosolic part; and (molecular function) oxidoreductase activity (Table S12).

In ER negative tumors that did not present downstaging, 39 gene sets were identified in the GSEA analysis (FDR < 0.25) including two KEGG pathways, related with arginine and proline metabolism, as well as the following four GO gene sets (FDR < 0.1): (cellular component) intermediate filament cytoskeleton (including *KRT6A*, *KRT1*, *KRT19*, *KRT18*, and *KRT31*); synaptic vesicle; intermediate filament; and (molecular function) neuropeptide receptor activity (Table S13).

The next step was to test our samples for the predictive value of a gene profile that was previously shown to classify samples according to tumor response. It consisted of a 50-gene signature [7], reflecting the activation state of the tumor stroma, which was related with poor response (characterized as non-pathological complete response to anthracycline based chemotherapy) in ER negative breast cancer. We then used this list of genes to perform a hierarchical clustering analysis of 15 ER negative samples (IMH of FFPE samples). Expression of these 50 genes, however, could not correctly classify samples according to response to chemotherapy, because it was rather homogeneous across samples. Expression of a group of genes, including FAP, fibroblast activation protein, and alpha; three types of collagen, *COL3A1*, *COL10A1*, *COL5A2*; two metalloproteases, *MMP11* and *MMP14*, among seven others was relatively high among most samples. In addition, a relatively moderate and high expression of *PRSS11* (or *HTRA1*, HtrA serine peptidase 1), *COL1A2*, *MMP2*, *TGFB3*, *SPARC* and *DCN* (decorin), as well as a relatively low expression of another group of genes, including *SNAI2* and *THBS2* (Figure S4) was observed in most samples.

Finally, we searched our samples for a gene profile that might differentiate the stromal cells, according to disease outcome, defined as recurrence of disease or death, however, we could not identify a stromal prognostic signature associated with this outcome.

We have also tested whether a previously identified stromal gene profile, associated with disease outcome, named stroma-derived prognostic predictor, SDPP [6], could predict recurrence or death of our patients. The expression of 24 genes, out of the whole list of 26 genes described in SDPP (excluding *TRBV5-4* and *C21orf34*, which were not tested in our samples), however, could not cluster the samples, according to disease outcome (Figure S5).

## 4. Discussion

We directly analyzed breast cancer stromal cells to identify a predictive signature of response to neoadjuvant chemotherapy. Using microdissected stromal cells, from samples collected before the start of neoadjuvant chemotherapy, we could identify eight genes more expressed in tumors presenting downstaging, including three genes involved in abnormal cytotoxic T cell physiology, such as *TOX*, *LY75*, and *SH2D1A*. In addition, gene sets correlated with tumor downstaging were mainly related with immune system pathways.

In our study, genes, such as *GATA3*, *ERBB4*, *RET*, *NAT1*, and *TFF3*, typically more expressed in ER positive tumor samples, were also more expressed in stromal cells from ER positive as compared with ER negative tumors (defined by immunohistochemistry of malignant cells) [20,21,22]. This result may reflect estrogen responsiveness, mediated by ER expression in stromal fibroblasts [23], as well as some degree of contamination of the samples of microdissected stromal cells with malignant cells (ER positive or ER negative). Among the genes more expressed in ER negative tumors, there was *HSD17B2*, which codes for an enzyme involved in metabolizing androgens and estrogen to less active metabolites, further indicating that the estrogen pathway is not critical in these tumors.

In accordance with previous reports in prostate cancer fibroblasts [24], we observed that AR is expressed in breast cancer stromal cells. Although there are indications that high AR levels may be associated with response to chemotherapy [18], stromal AR mRNA was not differentially expressed in DS vs. NDS samples.

We could identify a panel of differentially expressed genes between young (less than 41 years) and older patients. This was rather expected because differences in expression levels of dermal fibroblast from young and old human beings had already been described [25]. In addition, menopause is associated with senile involution of the breasts, characterized by progressive changes in the assemblage of the mammary parenchyma and reduced breast density, reflecting stroma and adipose tissue replacement of the alveoli [26]. However, in the present group of samples, we could not identify gene sets associated with particular pathways, or a differential profile using higher age cutoffs of 45 and 50 years (data not shown).

In stromal cells from responsive tumors, genes such as *PTCHD1*, *LY75*, *SH2D1A*, and *TOX* were more expressed, while *H2AFJ* was less expressed. More interestingly, a group of four pairs of genes were sufficient to classify samples according to response which included: *PTCHD1/PDXDC2P, LOC100506731/NEURL4*, *SH2D1A/ENST00000478672*, and *TOX/H2AFJ* with a high confidence.

*H2AFJ* encodes a member of the histone H2A super family, that may be overexpressed in breast cancer samples through gene amplification and considered a putative breast cancer oncogene [27]. Consistent with the *H2AFJ* overexpression observed in non-responsive breast cancer samples, in colorectal cancer cell lines, *H2AFJ* was described as a mediator of chemoradiation resistance (Wang et al., 2019) [28]. *PTCHD1* codes for a patched-related protein, structurally similar to the Hedgehog (Hh) receptors PTCH1 and PTCH2. PTCHD1 exhibits biochemical activity in Hh-dependent processes similar to the inhibitory effect of PTCH1 and PTCH2 on Gli-dependent transcription and may be expressed in mammary glands [29]. PTCH1 directly inhibits smoothened (SMO) and in a mouse model of pancreatic cancer, SMO inhibition may facilitate chemotherapy delivery and extend survival by depleting tumor-associated stromal tissue [30]. This mechanism might also be involved in chemotherapy induced downstaging in breast cancer samples.

*SH2D1A* and *TOX* were previously shown to be upregulated in germinal center T helper cells as compared with other CD4+ T cell subsets [31]. However, TOX overexpression in CD8+ tumor-infiltrating lymphocytes was also related with tumor immunosuppressive microenvironment, T cell exhaustion, and tumor persistence (Scott et al., 2019) [32]. In turn, *LY75 (CD205)* is expressed at relatively high levels on myeloid blood dendritic cells and monocytes and plays a role in endocytic uptake of antigen and presentation to lymphocytes via MHC class II molecules [33,34]. The present results may reveal that a complex interrelationship among specific players of the immune response take place in responsive tumors to AC-T.

In addition, various immune response gene sets were positively correlated with tumor downstaging. These gene sets comprehended genes such as *CD24*, that modulates B cell activation responses, *CD3D* and *CD3E*, which are part of the T-cell receptor/CD3 complex, that couples antigen recognition to several intracellular signal-transduction pathways; *CD7*, found on thymocytes and mature T cells and playing an essential role in T cell interactions; *CD40LGT*, expressed on the surface of T cells, regulating B cell function by engaging CD40 on the B cell surface; *CD74* that associates with class II major histocompatibility complex (MHC) and is an important chaperone that regulates antigen presentation for immune response; *CD79B*, the B lymphocyte antigen receptor, a multimeric complex that includes the antigen-specific component, surface immunoglobulin (Ig), necessary for expression and function of the B-cell antigen receptor; *CD83*, involved in the regulation of antigen presentation; CD96 that plays a role in the adhesive interactions of activated T and NK cells during the late phase of the immune response, as well as function in antigen presentation.

In an exploratory analysis, we individually analyzed the expression of some classical markers of tumor immune response. We observed a higher expression of CD8, CD3E, and CD247 (CD4Z) in responsive tumors. In contrast, a significant correlation between pCR and higher prechemotherapy infiltration by CD3, CD4, and CD20 has been previously described [35].

Interestingly, gene sets enriched in immune-related genes were more strongly correlated with tumor downstaging in ER negative tumors (more stringent FDR *q*-values). In accordance, it has been previously shown, in a cohort of triple negative tumors and HER2 positive tumors, that an increased percentage of stromal T infiltrating lymphocytes, as well as expression of immune activating and immunossupressive genes, are positively correlated to response to neoadjuvant chemotherapy [10]. In our study, immune response gene sets were also correlated with tumor downstaging in ER positive tumors, such as “KEGG antigen processing and presentation”, containing genes such as *CD74*, *CD8A*, and *CD8B*, and various genes of the HLA complex, as well as GO biological process gene set such as “regulation of T cell activation”, comprehending *ZAP70*, *CD24*, *CD3E*, *LCK*, *IL21*, among others.

There was not a clear pattern of gene sets associated with tumor resistance, however, stromal cells from both ER positive as well as ER negative tumors, which were enriched in cytoskeleton transcripts, might be associated with tumor non-downstaging. In addition, in ER positive tumors, cell adhesion, as well as KEGG ECM receptor interaction, might also be involved in tumor resistance.

In the group of estrogen receptor negative samples, we evaluated whether a previously reported stroma-related gene signature would predict resistance [7], however, in the present series of microdissected stromal cells, this gene profile could not separate resistant from responsive samples (pathological complete response or tumor downstaging), and a common pattern of gene expression could be distinguished. The high expression of various collagens and metalloproteases confirms the mesenchymal origin of these samples, whereas expression of *FAP*, a serine protease, indicates the reactive nature of nontransformed tumor stroma. In a previous work, we found that *PRSS11* was more expressed in responsive tumors to doxorubicin-based chemotherapy, however, in stromal cells from the current ER negative tumors, it was not differentially expressed between responsive and resistant samples [2].

We could not detect a stromal signature associated with prognosis in our whole series of samples. In addition, using a previously reported prognostic stromal cell signature identified in ER negative samples, named stroma-derived prognostic predictor (SDPP), we could not predict the clinical outcome for patients bearing ER negative tumors in the present series [6]. It is interesting to observe that, in the previous study, tumor stroma samples from the good-outcome cluster overexpressed a distinct set of immune-related genes. In the present series of stromal cells, however, gene sets of immune-related genes were mainly correlated with tumor downstaging.

Strengths of our study include the specific evaluation of microdissected stromal cells from patients with locally advanced breast cancer, who underwent the same neoadjuvant chemotherapy regimen and the identification of pairs of genes that might classify tumors according to response to neoadjuvant chemotherapy. Limitations include a relatively small number of patients and the intensive labor associated with tumor microdissection, that complicates the use in the clinical practice.

## 5. Conclusion

In summary, tumor stromal cells, which are non-malignant cells, represent an interesting option to evaluate response to neoadjuvant chemotherapy. In locally advanced breast cancer, stromal cells may present specific features of immune response that may be associated with chemotherapy response.

## Figures and Tables

**Table 1:** Characteristics of patients. Abbreviations: HT, histological type; D, ductal; L lobular; o, other; IS, in situ; Tdim, tumor dimension; preCT, prechemotherapy; and postCT postchemotherapy; T1mi, T1 microscopic; −, negative; +, positive. Estrogen receptor (ER) and progesterone receptor (PR) were considered positive if ≥1% malignant cells were stained. ND, not done.

*Id*	*Age (y)*	*HT*	*ER*	*PR*	*HER2*	*Ki67 (%)*	*T dim preCT (cm)*	*T dim postCT (cm)*	*ypTN (postCT)*
52	≤40	D	+	+	−	5	6.0	3.5	ypT2N0
84	>40	L	+	−	−	5	8.0	0.0	ypT0N0
67	>40	D/L	+	+	−	10	8.3	18.0	ypT4N3
20	≤40	D	+	+	−	10	6.0	6.0	ypT4N2
71	≤40	D/IS	+	+	−	10	7.6	4.5	ypT2N3
66	>40	D	+	+	−	60	11.0	0.8	ypT1bN0
68	>40	D	+	+	−	80	8.0	5.4	ypT3N2
33	>40	D	+	+	−	20	6.0	0.6	ypT1bN2
64	≤40	D	+	+	−	20	8.0	1.8	ypT1cN1
54	>40	D	+	+	−	20	9.0	8.3	ypT3N0
19	>40	D	+	+	−	20	7.0	8.0	ypT3N2
37	≤40	D	+	+	−	20	6.5	6.5	ypT3N1
58	≤40	D	+	−	−	30	10.0	11.0	ypT4N3
7	>40	D	+	+	−	30	7.8	1.5	ypT1cN3
79	≤40	D	+	+	−	70	5.2	0.0	ypT0N0
9	>40	D	−	+	−	70	4.0	4.4	ypT2N0
6	>40	D	−	+	−	80	6.0	7.8	ypT4N2
73	≤40	D	+	+	−	80	8.0	5.8	ypT3N2
63	>40	D	+	+	−	90	6.5	0.6	ypT1bN0
80	≤40	D	+	+	−	90	6.1	2.8	ypT4Nx
81	≤40	D	+	−	ND	100	6.4	5.0	ypT2N1
30	>40	L	+	+	−	ND	6.0	5.5	ypT3N1
56	≤40	D	+	+	+	ND	14.0	6.8	ypT3N1
62	>40	D	+	+	−	10	6.0	2.0	ypT1cN0
55	>40	D	+	+	−	10	5.5	6.3	ypT4N2
99	>40	D	+	+	+	10	12.0	0.8	ypT4N0
17	>40	D	+	−	+	20	8.0	9.0	ypT4N2
69	≤40	D	+	−	−	90	5.5	9.0	ypT3N3
18	>40	D	+	−	−	90	4.0	1.7	ypT1N2
11	>40	D	+	+	+	ND	7.0	2.2	ypT2N2
72	>40	D	+	+	+	ND	5.5	0.0	ypT0N1
59	>40	D	−	−	+	60	6.0	5.8	ypT3N1
49	>40	D	−	−	+	90	8.0	0.0	ypT1miN0
60	≤40	D	−	−	-	ND	3.5	0.0	ypT0N0
44	≤40	D	−	−	+	100	5.4	2.0	ypT1cN0
70	>40	D/O	−	−	−	40	5.1	4.5	ypT2N2
75	>40	D	−	−	−	60	7.0	7.5	ypT4N3
51	≤40	D	−	−	+	80	8.9	4.5	ypT4N1
38	>40	D	−	−	−	90	5.2	1.0	ypT1bN0
36	≤40	D	−	−	−	90	6.5	4.0	ypT4N3
76	>40	D	−	−	−	90	8.5	0.0	ypTisN0
45	>40	L	−	−	−	ND	7.5	4.0	ypT4N3
13	≤40	D	−	−	−	ND	6.5	4.2	ypT2N1
61	>40	D	−	−	−	100	5.5	0.3	ypT1aN0

**Table 2:** Gene sets associated with tumor downstaging. Gene expression of all 44 stromal cell samples were analyzed through gene set enrichment analysis (GSEA) (FDR < 0.01). Gene sets and rank ordering of distinct pathway members, associated with the phenotype downstaging, are shown.

GO Gene Sets (Biological Process)	Genes (CORE ENRICHMENT)
ADAPTIVE_IMMUNE_RESPONSE_GO_0002460	FOXP3 CD74 CRTAM C2 TRAF2 MAP3K7 TNFSF13 SOCS5 TRAF6 IL18 TLR8 EBI3 PTPRC CD40LG
ADAPTIVE_IMMUNE_RESPONSE	FOXP3 CD74 CRTAM C2 TRAF2 MAP3K7 TNFSF13 SOCS5 TRAF6 IL18 BCL10 TLR8 EBI3 PTPRC CD40LG
POSITIVE_REGULATION_OF_LYMPHOCYTE_ACTIVATION	CD47 LCK NCK1 CD24 CD3E ICOSLG TNFSF13 ZAP70 SOCS5 IL18 EBI3 PTPRC SIRPG
REGULATION_OF_IMMUNE_SYSTEM_PROCESS	FOXP3 APOBEC3G CD47 LCK TGFB2 NCK1 LAX1 CD24 CD3E ICOSLG CRTAM C2 TRAF2 MAP3K7 TNFSF13 ZAP70 SOCS5 TRAT1 TRAF6 IL18 EREG UBE2N TLR8 EBI3 PTPRC SIRPG NCR1 FYN NFAM1 LAT2 INHBA CD28
POSITIVE_REGULATION_OF_IMMUNE_SYSTEM_PROCESS	CD47 LCK TGFB2 NCK1 CD24 CD3E ICOSLG CRTAM C2 TRAF2 MAP3K7 TNFSF13 ZAP70 SOCS5 TRAT1 TRAF6 IL18 EREG UBE2N TLR8 EBI3 PTPRC SIRPG FYN NFAM1 LAT2 CD28
POSITIVE_REGULATION_OF_T_CELL_ACTIVATION	CD47 LCK NCK1 CD24 CD3E ICOSLG ZAP70 SOCS5 IL18 EBI3 PTPRC SIRPG
IMMUNE_RESPONSE	LY75 FOXP3 APOBEC3G IL15 CTSS TRIM22 TLR7 PTGER4 POU2AF1 PRKRA CD74 TGFB2 IL10RB DEFB1 TAPBP LAX1 CXCL13 HLA-DRB3 FYB BLNK NFIL3 CD96 SKAP1 CRTAM C2 TRAF2 IRF8 CD83 CTSC TCF7 MAP3K7 TNFSF13 CHUK ZAP70 IL2 YTHDF2 SOCS5 TRAT1 CCL5 TRAF6 IL6 AIM2 IL18 CCL25 BCL10 IKBKAP EREG LCP2 CXCR4 OPRK1 UBE2N CCL20 TNFAIP1 CCL2 LTB4R TLR8 CEBPB WAS CD164 SECTM1 GTPBP1 EBI3 CD7 TCF12 CD79B IL2RG GEM PTPRC GZMA CCR5 NCR1 CCL23 GPR65 FYN CD40LG XBP1 DPP4 CCR2 MAP4K2 APOA1 NFAM1 NCF4 LAT2
_S_TRANSITION_OF_MITOTIC_CELL_CYCLE	CUL2 CUL1 GFI1 CDKN2A LATS2 CDKN2C PPP6C ACVR1 CDKN1B INHBA CDCA5 CDKN1A GSPT1 ACVR1B CDKN2D RCC1
DNA_DEPENDENT_DNA_REPLICATION	GTPBP4 MSH5 RFC4 RAD17 CCDC88A RFC3 MSH6 MSH2 PRIM1 POLA1 TSPYL2 RFC1 PRIM2 GMNN POLB EREG HMGB2 CDK2AP1 REV3L S100A11 EXO1 NBN CDC6 MLH1 RPAIN
T_CELL_ACTIVATION	FOXP3 CD47 LCK NLRC3 NCK1 LAX1 CD24 CD3E ICOSLG CRTAM NHEJ1 ZAP70 IL2 SOCS5 IL18 EBI3 CD7 PTPRC SIRPG
APOPTOSIS (APOPTOSIS_GO)	CASP1 BAX NFKB1 IRF1 TNFRSF21 IRF4 GZMB FAS BID NFKBIA CASP3 TRAF2 CASP7 CHUK BIRC2 MDM2 TP53 TRAF3 TNF NFKBIE FASLG CASP4 APAF1 BIRC3 CASP6 TRAF1 CYCS
REGULATION_OF_IMMUNE_EFFECTOR_PROCESS	FOXP3 APOBEC3G CRTAM TRAF2 MAP3K7 TRAF6 PTPRC NCR1
LYMPHOCYTE_ACTIVATION	FOXP3 CD47 LCK NLRC3 NCK1 LAX1 CD24 CD3E ICOSLG CRTAM TPD52 NHEJ1 TNFSF13 ZAP70 IL2 SOCS5 IL18 EBI3 CD7 PTPRC SIRPG CD40LG NFAM1 LAT2 INHBA CD28 CD3D
POSITIVE_REGULATION_OF_CYTOKINE_PRODUCTION	TRAF2 NOD2 MAP3K7 TRAF6 EREG IFNG ATP6AP2 CD40LG
POSITIVE_REGULATION_OF_MULTICELLULAR_ORGANISMAL_PROCESS	CD47 LCK TGFB2 NCK1 CD24 CD3E ICOSLG CRTAM C2 TRAF2 NOD2 MAP3K7 TNFSF13 ZAP70 SOCS5 TRAT1 TRAF6 IL18 EREG IFNG UBE2N TLR8 EBI3 PTPRC ATP6AP2 SIRPG BMP4 FYN CD40LG NFAM1 LAT2 CD28 MC4R

## Supplementary Materials

The following are available online at https://www.mdpi.com/2073-4409/8/12/1566/s1. Figure S1: Microphotography of eosin stained breast cancer sample before and after selection of stromal cells. (A) Breast cancer sample submitted to laser capture microdissection, using ArcturusXTTM. (B) Selection of stromal cells (inside the black line). (C) Tumor slice after removal of stromal cells. (D) Microdissected stromal cells. Figure S2: Unsupervised hierarchical clustering of stromal cells from patients categorized as ≤40 and > 40 years. Two branches were identified with high confidence, one including 17/44 samples from patients ≤ 40 years and the other including all samples from patients > 40 years. Upper box: age ≤40 marked in red. Green - red scale bar on the top: >40/ ≤40 years. Figure S3: Enriched networks in stromal cells according to tumor downstaging. Data derived from gene expression was investigated for enriched networks, using Ingenuity Pathway Analysis, IPA (Qiagen). Top diseases and functions enriched in the gene list considered differentially expressed, were tissue morphology, cancer and developmental disorder. This gene list included H2AFJ, LY75, PTCHD1, RASGRF1, SH2D1A, SH3GL3, TOX (painted gray on the figure). Figure S4: Unsupervised hierarchical clustering of stromal cells from tumors categorized as ER negative, by immunohistochemistry reaction, using a 50-gene signature [7], previously described as a predictor of poor response to chemotherapy. Expression of these 50 genes could not correctly classify samples according to complete pathological response (PCR) or tumor downstaging (DS) (PCR: samples 60, 76; DS: 38, 61, 49). Figure S5: Unsupervised hierarchical clustering of stromal cells, using a previously identified stromal gene profile associated with disease outcome, named stroma-derived prognostic predictor, SDPP [6]. Expression of 24 genes, out of the whole list of 26 genes described in SDPP (excluding TRBV5-4 and C21orf34, which were not present in the list of genes tested in the present study) was used to cluster samples. This gene profile could not cluster the samples, according to disease outcome (recurrence or death).Table S1: Functional enrichment analysis of the top 50 highly expressed genes in stromal cells from microdissected breast cancer samples (Toppgene Suite). Table S2: Expression of epithelial cell markers in stromal cells obtained from luminal and triple negative breast cancer samples. Table S3: Sequences differentially expressed between stromal cells obtained from ER positive and ER negative tumors (according to immunohistochemistry from FFPE tumor samples). Fc: fold change (ER positive/ ER negative). Table S4: Correlation between mRNA expression of hormone receptors in stromal cells of BC samples. Table S5: Sequences differentially expressed between stromal samples from patients aged > 40 years or ≤40. Fc: fold change (> 40 years/ ≤40 years). Table S6: Sequences differentially expressed between stromal cells obtained from tumors presenting or not presenting downstaging after chemotherapy. (DS; NDS: non-downstaging). Gene expression of microdissected stromal cells was evaluated in Agilent SurePrint G3 8x60K slides, applying SAM test (FDR 17). Fc: fold change (DS/NDS). Table S7: GO gene sets associated with tumor downstaging. Gene expression of all 44 stromal cell samples were analyzed through GSEA, (gene set enrichment analysis). Gene set name, collection, FDR q value and genes in rank ordering of distinct pathway members, associated with the phenotype downstaging, are shown. Table S8: GO gene sets associated with tumor non-downstaging. Gene expression of all 44 stromal cell samples were analyzed through GSEA, (gene set enrichment analysis). Gene set name, collection, FDR q value and genes in rank ordering of distinct pathway members, associated with the phenotype non-downstaging, are shown. Table S9: Expression of hormone receptors, lymphocyte markers and epithelial markers in stromal cells from downstaging (DS) and non-downstaging (NDS) samples. Table S10: GO gene sets associated with tumor downstaging in ER positive tumors, (by immunohistochemistry). Gene expression of stromal cells obtained from ER positive 29 tumors were analyzed through GSEA (gene set enrichment analysis). Gene set name, collection, FDR q value and genes in rank ordering of distinct pathway members, associated with the phenotype downstaging, are shown. Table S11: GO gene sets associated with tumor downstaging in ER negative tumors, (by immunohistochemistry). Gene expression of stromal cells obtained from ER negative 15 tumors were analyzed through GSEA (gene set enrichment analysis). Gene set name, collection, FDR q value and genes in rank ordering of distinct pathway members, associated with the phenotype downstaging, are shown. Table S12: GO gene sets associated with tumor non-downstaging in ER positive tumors, (by immunohistochemistry). Gene expression of stromal cells obtained from ER positive 29 tumors were analyzed through GSEA (gene set enrichment analysis). Gene set name, collection, FDR q value and genes in rank ordering of distinct pathway members, associated with the phenotype non-downstaging, are shown. Table S13: GO gene sets associated with tumor non-downstaging in ER negative tumors (by immunohistochemistry). Gene expression of stromal cells obtained from ER negative 15 tumors were analyzed through GSEA (gene set enrichment analysis). Gene set name, collection, FDR q value and genes in rank ordering of distinct pathway members, associated with the phenotype non-downstaging, are shown.
