# Supplementary material for: Stromal Cell Signature Associated with Response to Neoadjuvant Chemotherapy in Locally Advanced Breast Cancer
Source: Cells. 2019 Dec 4;8(12):1566. doi: 10.3390/cells8121566 (PMC6953077; doi:10.3390/cells8121566)
Supplement: Supplementary file 1 [file cells-08-01566-s001.zip › cells-612497-supplementary/Supplementary Figures.docx]

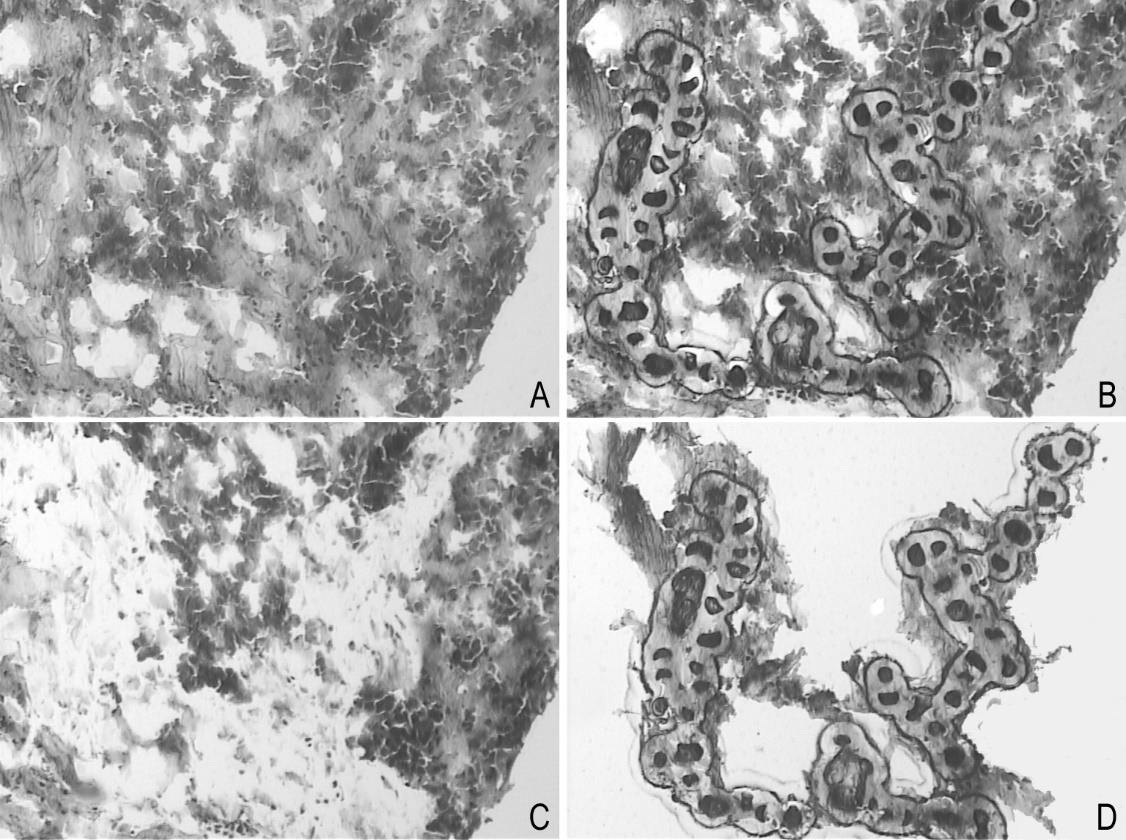


Figure S1: Microphotography of eosin stained breast cancer sample before and after selection of stromal cells. (A) Breast cancer sample submitted to laser capture microdissection, using ArcturusXTTM. (B) Selection of stromal cells (inside the black line). (C) Tumor slice after removal of stromal cells. (D) Microdissected stromal cells.


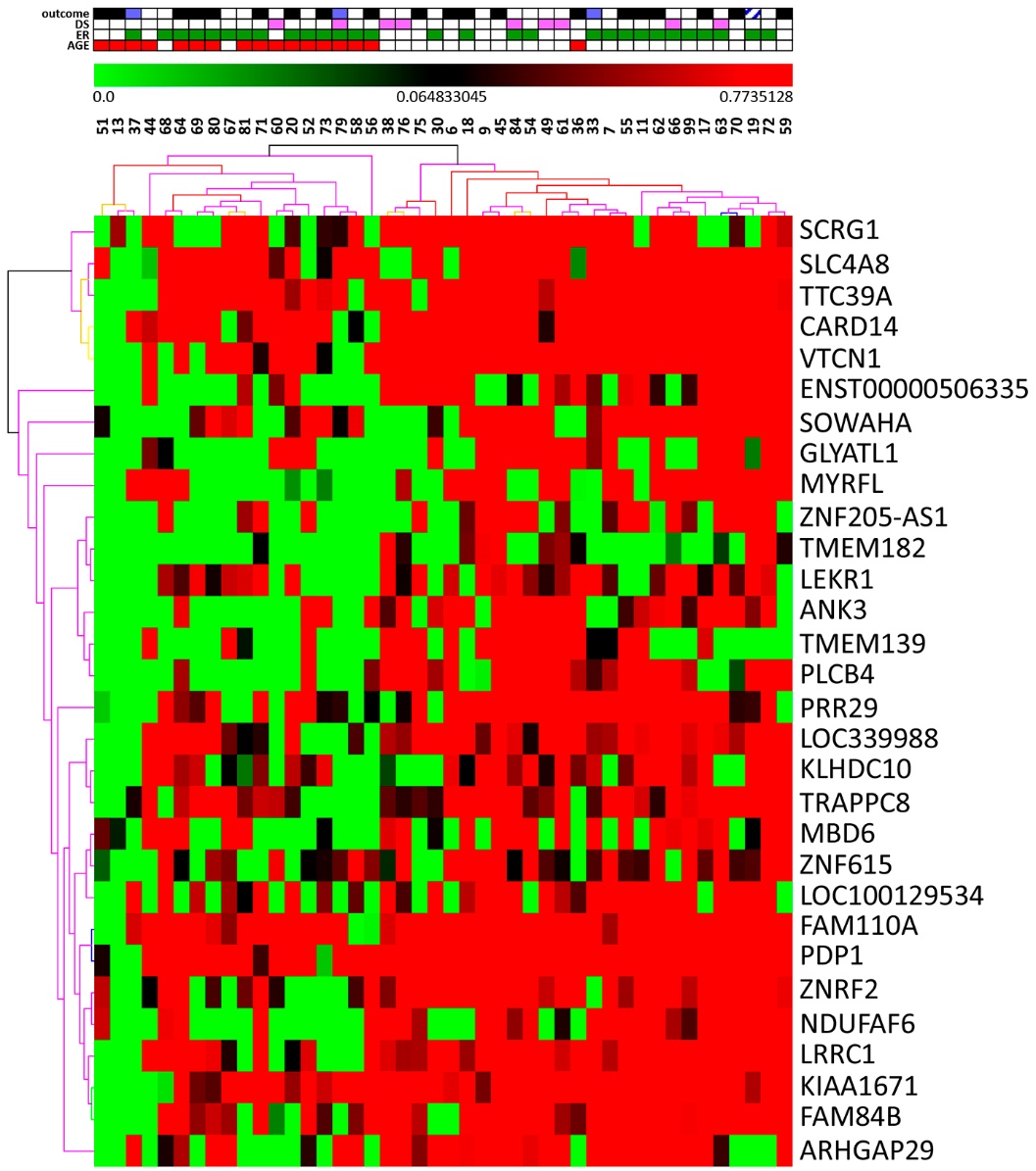


Figure S2: Unsupervised hierarchical clustering of stromal cells from patients categorized as ≤40 and > 40 years. Two branches were identified with high confidence, one including 17/44 samples from patients ≤ 40 years and the other including all samples from patients > 40 years. Upper box: age ≤40 marked in red. Green - red scale bar on the top: >40/ ≤40 years.


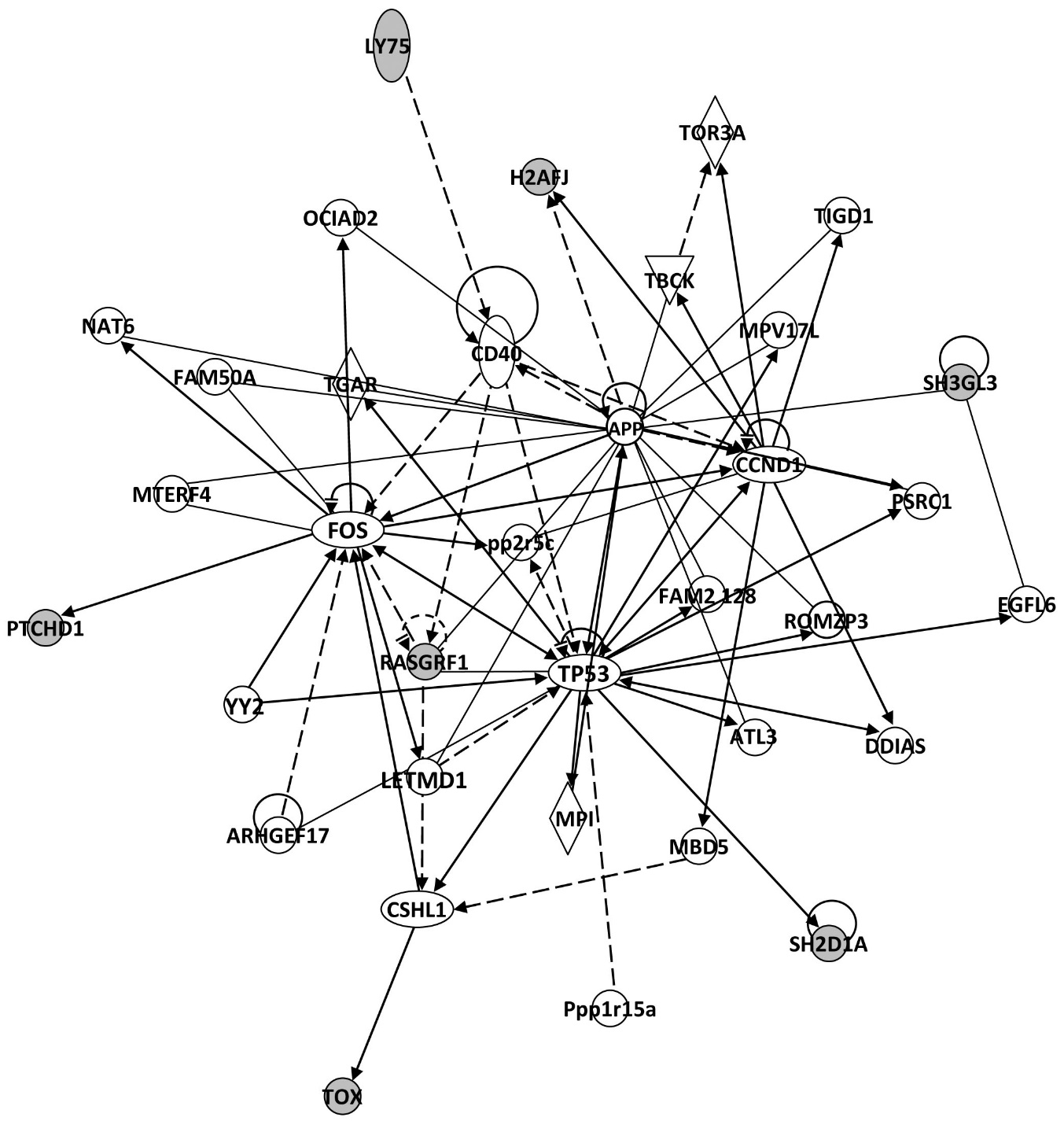


Figure S3: Enriched networks in stromal cells according to tumor downstaging. Data derived from gene expression was investigated for enriched networks, using Ingenuity Pathway Analysis, IPA (Qiagen). Top diseases and functions enriched in the gene list considered differentially expressed, were tissue morphology, cancer and developmental disorder. This gene list included H2AFJ, LY75, PTCHD1, RASGRF1, SH2D1A, SH3GL3, TOX (painted gray on the figure).


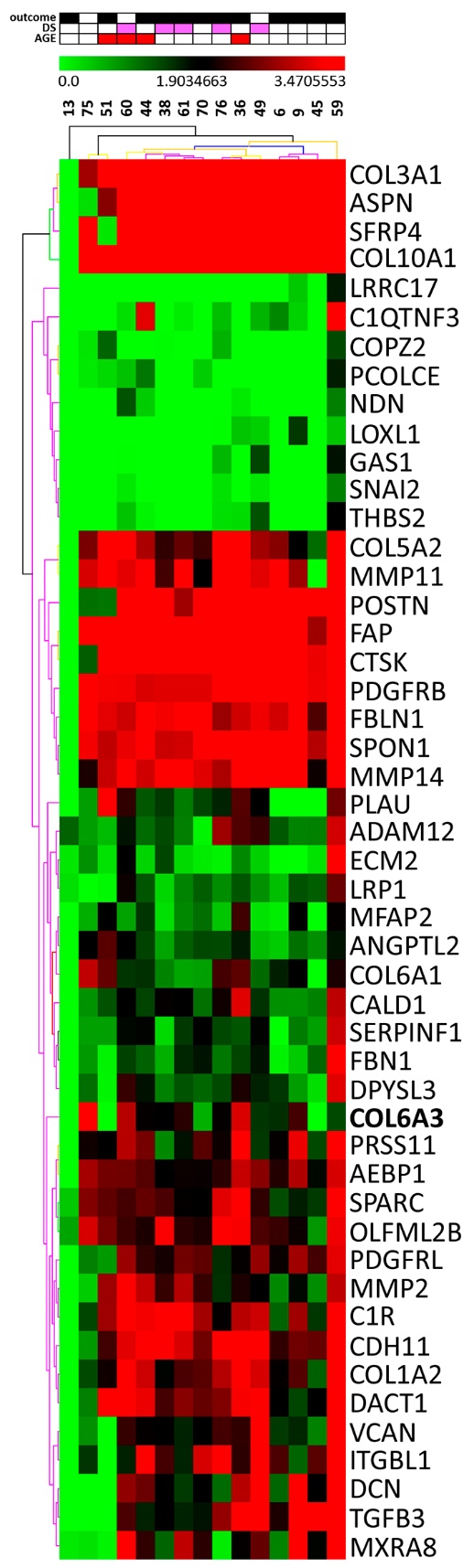


**Figure S4.** Unsupervised hierarchical clustering of stromal cells from tumors categorized as ER negative, by immunohistochemistry reaction, using a 50-gene signature [7], previously described as a predictor of poor response to chemotherapy. Expression of these 50 genes could not correctly classify samples according to complete pathological response (PCR) or tumor downstaging (DS) (PCR: samples 60, 76; DS: 38, 61, 49).


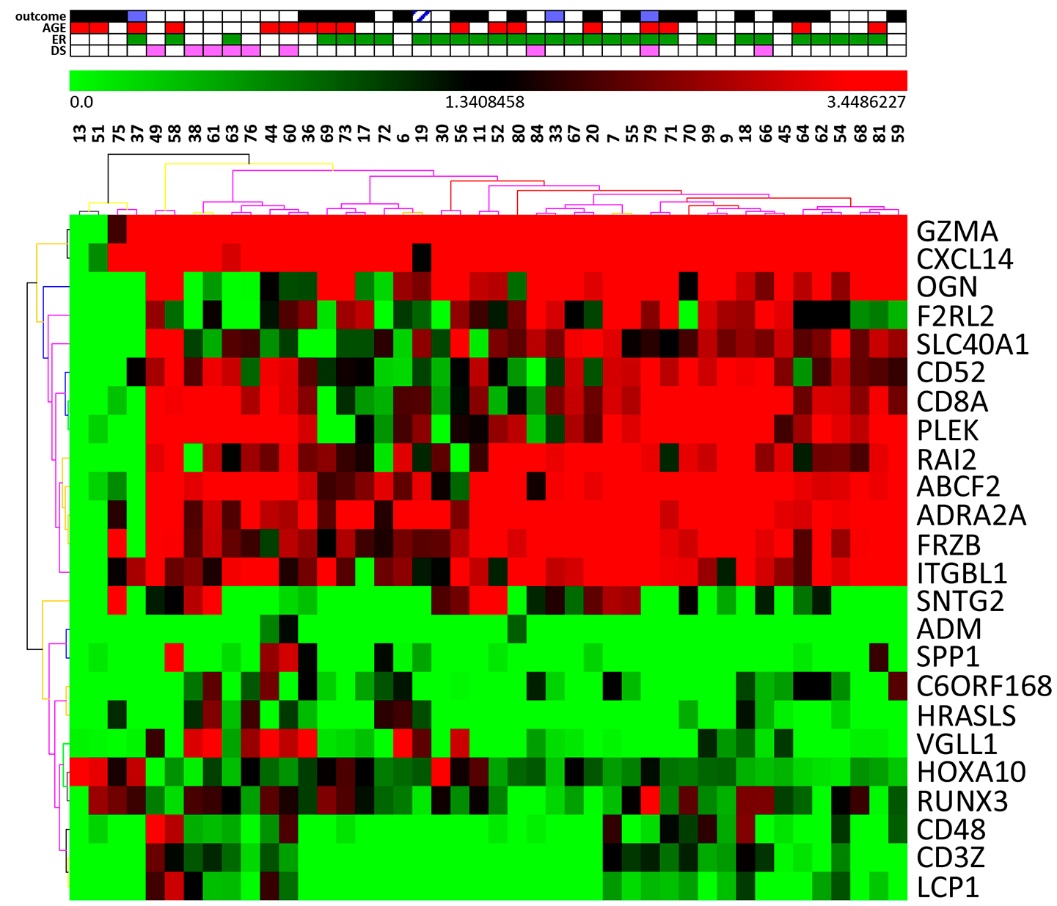


**Figure S5.** Unsupervised hierarchical clustering of stromal cells, using a previously identified stromal gene profile associated with disease outcome, named stroma-derived prognostic predictor, SDPP [6]. Expression of 24 genes, out of the whole list of 26 genes described in SDPP (excluding TRBV5-4 and C21orf34, which were not present in the list of genes tested in the present study) was used to cluster samples. This gene profile could not cluster the samples, according to disease outcome (recurrence or death).
