# Supplementary material for: Stromal Cell Signature Associated with Response to Neoadjuvant Chemotherapy in Locally Advanced Breast Cancer
Source: Cells. 2019 Dec 4;8(12):1566. doi: 10.3390/cells8121566 (PMC6953077; doi:10.3390/cells8121566)
Supplement: Supplementary file 1 [file cells-08-01566-s001.zip › cells-612497-supplementary/Supplementary Methods.doc]

**Supplementary Methods**

**Tumor immunohistochemistry**

Estrogen receptor (ER) and progesterone receptor (PR) expression were evaluated using anti-estrogen receptor alpha rabbit monoclonal antibody SP1 (Thermo Fisher Scientific, Walthan, MA, USA) and CONFIRM anti-progesterone receptor rabbit monoclonal antibody (Roche AB, Christian Sundberg, Stockholm, Sweden), respectively, and were considered positive if ≥ 1% malignant cells were stained. HER2 immunoexpression was evaluated through HercepTest (Dako Agilent Patology solutions, Santa Clara, CA, USA) and/or FISH (HER2 FISH pharm Dx ™) and was considered hyperexpressed or amplified, according to the manufacturer´s interpretation guides. Ki67 expression was evaluated using monoclonal mouse anti-human Ki-67 Antigen, Clone MIB-1 (Dako Agilent Pathology Solutions).

**Stromal cell selection and Microarray**

At first, fresh frozen tumor fragments were cut in 10 µm slices and immobilized in slides. One of the slides was stained with HE for analysis and samples without infiltrating stroma were not considered for analysis. The other tumor slides were dehydrated, using Histogene® Refill Kit (Thermo Fisher Scientific), and eosin stained. Afterwards intratumoral stromal cells were visualized and captured by the transfer film bounded to the lower surface of a cap (CapSure HS LCM,Thermo Fisher Scientific), through infrared pulses, shot by Pix Cell II Arcturus Laser Capture Microdissection (Thermo Fisher Scientific) (LCM) (Supplementary Fig 1). The captured area, mainly comprised fibroblasts, which were sometimes accompanied by imune cells. Tumor nests and blood vessels were avoided. A lysis solution (12 μl) was added to the cap for extraction of a cell lysate, which was then placed in a preconditioned RNA purification column. After spinning, the column was washed, with wash buffer, and spun, twice, and eluted in 30 μl elution buffer (Arcturus PicoPure RNA isolation, Thermo Fisher Scientific).

At least 100 pg total RNA, recovered from the columns, was then mixed with spike B. In addition, a Universal Reference RNA (Stratagene California, La Jolla, CA, USA), composed of high-quality total RNA from 10 human, 11 mouse and 14 rat cell lines, which acts as a consistent control for standard data set comparisons in competitive microarray assays was mixed with spike A. Spikes A and B (Agilent RNA Spike-In Kit, Agilent Technologies) presents different composition of synthetic polyadenylated transcripts, derived from the Adenovirus E1A transcriptome, that are premixed at various ratios, and in microarray experiments are used as amplification and hybridization quality control.

Both total RNA from samples and Universal Reference RNA were linearly amplified, using two-round RiboAmp HS^Plus^2-round (Thermo Technologies) and Low Input Quick Amp kit (Agilent Technologies) protocols, which were adapted to optimize the amplification for the small number of cells isolated by LCM, as described by Agilent Technologies. During this *in vitro* transcription, *s*amples of interest, as well as the Universal Reference, were labelled with cyanine 5 (CY5) or cyanine 3 (CY3), respectively. After fragmentation at 60^o^C during 10 minutes, samples were analyzed on a NanoDrop spectrometer (Thermo Fisher Scientific) to calculate the yield (concentration of cRNA (ng/µL) x 30 (elution volume)/1000= µg of cRNA) and specific activity (concentration of Cy3 or Cy5/concentration of cRNA) X 1000 = pMol Cy3 or Cy5 per µg cRNA).

Samples selected for the hybridization step were those presenting yields ≥0.825 µg of cRNA and specific activity ≥ 6 pMol Cy3 or Cy5 per µg cRNA, as recommended by Agilent Technologies. In our experiments samples and references presented values between 0.79 – 5.93 µg of cRNA, to yields, and 6.36 – 10.75 pMol Cy3 or Cy5 per µg cRNA, to specific activity. Competitive hybridization of each sample of interest-Cy5 and Universal Reference-Cy3 Fluorescent cRNAs (300 ng each) was performed in the SurePrint G3 8×60K slides (Agilent Technologies) at 60oC, during 20 hours. SurePrint G3 Human Gene Expression v3 Microarray features updated mRNA probes, optimized to the latest content sources, and long non-coding RNA (lncRNA) probes, to cover the catalog of lncRNAs from the LNCipedia 2.1 database. The accuracy and efficiency of hybridization was evaluated considering spike controls. After having washed the slides, the microarrays were scanned at 5-μm resolution with the Agilent Bundle Model B Microarray Scanner System (Agilent Technologies). Scanned image files were visually inspected for artifacts, and the fluorescence intensities were extracted and pre-processed with Agilent Feature Extraction software (v10.7.1). Normalization was done using GeneSpring GX12.1 software (Agilent Technologies).

Comparisons of expression levels were performed using MeV (MultiExperiment Viewer, version 4.5.1.) software applying SAM (Significance analysis of microarrays). Analysis was done using all sequences (without filtering). Unsupervised hierarchical clustering based on Euclidean distance and average linkage was used to verify association patterns. The reliability of the clustering was assessed by the Bootstrap technique using MEV.

Gene list enrichment analysis and candidate gene prioritization, based on functional annotations and protein interactions network, was performed using Toppgene suite, with FDR correction p value cut off ≤ 0.05 (available at [https://toppgene.cchmc.org/enrichment.jsp](http://h/)) [14].

Gene set enrichment analysis (GSEA) was used to identify whether predefined gene sets might associate with gene expression differences between phenotypes (available at http://software.broadinstitute.org/gsea/index.jsp). In this pairwise comparison, all genes are ranked based on signal-to-noise ratio. Then, the alternative hypothesis, that rank ordering of distinct pathway members is associated with a specific phenotype, is tested [13]. This methodology makes it possible to detect situations where all genes, in a predefined set, change in a small, but coordinated way. FDR<0.25 was considered significant. Some results are shown considering FDR q value < 0.1 or < 0.01, because even using more stringent cut offs, these lists comprehended at least 100 gene sets. GSEA collection was identified by searching google tool for gene set name.

Data derived from gene expression was also investigated for enriched networks, using Ingenuity Pathway Analysis, IPA (Qiagen).

**Stromal cells transcriptional profile (Supplementary fig 4)**

According to the result of supplementary fig 4, the classification of stromal cells samples according to tumor response, based on gene expression, based on Sample Percentiles and Populations Medians [11, 30]. This method compares two populations’ microarray medians. We denote by M_1_ and M_2_ the unknown medians of the microarrays of a specific marker. The result of the analysis is that of the confidence that the data provides about the parameters relation {M_1_ >M_2_}. The calculation of this confidence statement is done by comparing, for the two samples, the order statistics and respective percentiles levels. With the list of the confidence statements for all markers, over and under expressed markers pairs are chosen sequentially and an index based in a composition of the microarrays of these pairs are defined. The sequential algorithm is as follows:

i. Define the first pair as the two pairs with the most confidence; one over and other under expressed.  Our first pair of markers was *PTCHD1*; *PDXDC2P* .

ii. Divide the microarray measure of the first by the measure of the second.  After calculate this composition for the two samples compute the confidence as before. If the confidence is not high enough go to the next pair.

iii. The second pair with the most higher confidence was *LOC100506731*; *NEURL4*.  With the two pairs take the ratio of the product of the two over expressed and divide by the product of the two under expressed. With this new index calculate the confidence statement as before and if it is not high enough go to the next pair.

iv. Repeat the procedure to 3 pairs, the two above and the next pair with the most confident statement.  Calculate now a new index by multiplying the 3 over expressed divided by the product of the 3 most under expressed markers. If the confidence obtained was not enough go to the next pair.

v. Repeat the procedure to the fourth pair and again if necessary use another pair sequentially select new pairs up to obtain a satisfied confidence statement.

For our data set we could stop at the following 4 pairs of markers:

*PTCHD1*; *PDXDC2P*, *LOC100506731*; *NEURL4*, *SH2D1A*; *ENST00000478672*, *TOX*; *H2AFJ*
The final index allowed us to state that the confidence of Mi_1_>Mi_2_ is 99.8%.  Mi represents the median of the final index values.  This method is described in Marques & Pereira (2014) [13]. The figure illustrates the index values in the two populations and gives us a threshold to classify a new patient in one of the 2 population’s studies.
